# Observance of Sterilization Protocol Guideline Procedures of Critical Instruments for Preventing Iatrogenic Transmission of Creutzfeldt-Jakob Disease in Dental Practice in France, 2017

**DOI:** 10.3390/ijerph15050853

**Published:** 2018-04-25

**Authors:** Denis Bourgeois, Claude Dussart, Ina Saliasi, Laurent Laforest, Paul Tramini, Florence Carrouel

**Affiliations:** 1 Laboratory “Systemic Health Care”, EA4129, University Lyon, 69008 Lyon, France; denis.bourgeois@univ-lyon1.fr (D.B.); claude.dussart@univ-lyon1.fr (C.D.); inasaliasi@yahoo.com (I.S.); 2 Department of Public Health, Faculty of Dental Medicine, University of Lyon, 69008 Lyon, France; laurent.laforest@univ-lyon1.fr; 3 Department of Public Health, Faculty of Dental Medicine, University of Montpellier, 34090 Montpellier, France; paul.tramini@orange.fr

**Keywords:** dental assistants, dentists, sterilization, dental instruments, cross infection, infection control, transmissible diseases, Creutzfeldt-Jakob disease

## Abstract

Effective sterilization of reusable instruments contaminated by Creutzfeldt–Jakob disease in dental care is a crucial issue for public health. The present cross-sectional study investigated how the recommended procedures for sterilization were implemented by French dental practices in real-world settings. A sample of dental practices was selected in the French Rhône-Alpes region. Data were collected by a self-questionnaire in 2016. Sterilization procedures (*n* = 33) were classified into 4 groups: (1) Pre-sterilization cleaning of reusable instruments; (2) Biological verification of sterilization cycles—Monitoring steam sterilization procedures; (3) Autoclave performance and practitioner knowledge of autoclave use; (4) Monitoring and documentation of sterilization procedures—Tracking and tracing the instrumentation. Answers were provided per procedure, along with the global implementation of procedures within a group (over 80% correctly performed). Then it was verified how adherence to procedure groups varied with the size of the dental practice and the proportion of dental assistants within the team. Among the 179 questionnaires available for the analyses, adherence to the recommended procedures of sterilization noticeably varied between practices, from 20.7% to 82.6%. The median percentages of procedures correctly implemented per practice were 58.1%, 50.9%, 69.2% and 58.2%, in Groups 1, 2, 3 and 4, respectively (corresponding percentages for performing over 80% of the procedures in the group: 23.4%, 6.6%, 46.6% and 38.6%). Dental practices ≥ 3 dental units performed significantly better (>80%) procedures of Groups 2 and 4 (*p* = 0.01 and *p* = 0.002, respectively), while no other significant associations emerged. As a rule, practices complied poorly with the recommended procedures, despite partially improved results in bigger practices. Specific training regarding sterilization procedures and a better understanding of the reasons leading to their non-compliance are needed.

## 1. Introduction

In addition to traditional infectious diseases not only from the human immunodeficiency virus (HIV), but also from Gram-negative bacteria and Gram-positive bacteria, fungi, mycobacteria, tuberculosis and hepatitis B and C, preventing the risk of Creutzfeldt-Jakob disease (CJD) transmission is critical. Indeed, CJD is a fatal neurodegenerative disease that affects about one per million people per year [1,2]. The expected number of individuals suffering from the disease in 2020 is estimated to be between 80,000 and 136,000, according to different discordant sources and depending on the duration of incubation periods used [3]. Prions of CJD can be transmitted by various sources, including bovine spongiform encephalopathy, or iatrogenic routes such as growth hormones [4] or blood transfusions [5], or even dental care [6]. 

Though the risk of transmission during dental care is considered low, it cannot be overlooked, either [7,8]. The iatrogenic risk of a secondary infection related to an endodontic treatment when effective prion-inactivation procedures are not followed has been estimated to be between 3.4 and 13 cases per million treatments [9]. In oral health, the WHO regards human dental pulp as a “low-infectivity tissue” for prion diseases, given the presence of peripheral nerves [10], and the proximity of the nerves that make up the human dental pulp, with the central nervous system being a high-infectivity tissue [11,12]. These organisms can be transmitted in dental settings through direct contact with blood, oral fluids, or other patient materials, and indirect contact with contaminated objects (e.g., instruments, equipment, or environmental surfaces) [3]. Even if the risk is considered to be low, it is essential to observe good cleaning and sterilization practices as a means of preventing the iatrogenic transmission of prion proteins [13,14].

Given the high resistance of prions, conventional sterilization processes are inefficient in oral medicine [11]. Prions can be inactivated in a type B CLASS steam autoclave by standard EN 13060 as defined in the European Standard for Small Team Sterilisers at a temperature of 270 °F (134 °C) at 21 psi for 18 min [15,16,17,18]. The other cycles or types of autoclave do not make it possible to ensure the inactivation of the prions. By following the cleaning and sterilization recommendations for critical or semi-critical instruments, the iatrogenic risk of a secondary infection was practically zero [19]. 

Compliance with infection control recommendations and guidelines provides a safe working environment for dental health care personnel (DHCP) and their patients, to prevent or reduce the potential for infection transmission from patient to DHCP, from DHCP to patient, and from patient to patient [20,21,22]. Effective sterilization of instruments is crucial for prevention of transmission of communicable diseases, as well as delivery of quality dental care [23,24]. 

Changes in the guidelines in dental practices have evolved over time. The emergence of new data has resulted in the revision of the guideline standards with respect to the level of infectivity in tissues, the magnitude of the risk of infection for patients, and the definition of invasive procedures at risk of prion transmission, efficient protective processes against prions, processes of managing medical devices and instruments, and the conditions of their confinement and/or destruction. Although correct adherence to sterilization guidelines remains a high-priority challenge in terms of public health, poor compliance with standard practices has previously been documented [25,26]. No study specifically focusing on the successive steps of the sterilization of reusable instruments for preventing iatrogenic transmission of CJD in dental practice has been described in the literature.

The aim of this study was to evaluate, in accordance with the WHO guidelines, the observance of sterilization procedures for preventing iatrogenic transmission of Creutzfeldt-Jakob disease, including of reusable instruments in actual conditions of care in French dental practices.

## 2. Materials and Methods 

### 2.1. Sample

The Rhône-Alpes region comprises 6.3 million inhabitants—9.8% of the total French population—with 67% residing in urban areas. 3765 general dental practitioners (GDP) work in private practice, with very few working as employees (2%). Nearly two-thirds of practitioners (60.4%) practice their profession with other dentists, with only one-third reporting that they work alone. Dental practices with an average of at most one full-time equivalent (FTE) employee job represent more than half of the firms. More than a quarter had an average FTE of two and the remainder—almost a quarter—more than two. However, there are few practices with a total of more than five FTEs. As for the dentists’ employees, their staff is divided into receptionists, assistants, and dental assistants (DAs). Among dental assistant jobs, 58% of jobs are full-time and the rest are part-time (7860). Dental assistants perform sterilization under the responsibility of the GDP.

All private GDPs of all GPD members of the regional professional organization in the Rhône-Alpes region were eligible for the study, except those who exclusively practiced without DAs (40%), with a minimum of one DA per dental office, except those who exclusively practiced as orthodontists (<1%). The recruitment procedure and details about dental offices’ selection were based on the quota method: number of GDPs, number of DAs, number of dental units. The initial sample originally included 320, selected randomly. 

DAs were invited to participate by email, which included the procedure for completing and returning the questionnaire. The positive response rate was 56%. The final sample originally also included 179 selected DAs. The DAs were invited to participate in the study by email via a link to the survey. All community-based DAs working in private practice were eligible for the study. 

The respondents were assured that participation was voluntary. A single DA from each dental practice replied. The survey was conducted in 2017 as an anonymous online self-administered questionnaire. Implicit consent was assumed when a DA returned a completed questionnaire. Any data about the DAs who declined participation were collected. The questionnaires did not contain any identification data about respondents, and all collected data remained confidential throughout the study. The Regional Ethics Committee approved the study and it does not have any ethical issue or need for ethical approval code.

### 2.2. Development of the Questionnaire

The questionnaire included questions about thirty-three sterilization practices, distributed into four subscales of procedures: Group 1, Pre-sterilization cleaning of reusable instruments; Group 2, Biological verification of sterilization cycles—Monitoring steam sterilization procedures; Group 3, Autoclave performance and practitioner knowledge of autoclave use; Group 4, Monitoring and documentation of sterilization procedure—Tracking and tracing of the instrumentation.

The selection of the thirty-three practice items was based on the standard precautions adopted by the World Health Organization’s (WHO) “Health Service Executive Standards and Recommended Practices for Dental Services in a Local Decontamination Unit (LDU)”, HSE, 2012 (rev. edn 2014) applied by legislation in France via the French Ministry of Health [10,27,28].

Additional variables describing the dental practice as described above—number of dental units, number of dentists, number of DAs—were recorded.

The final version of the questionnaire was made up of 29 items. The questionnaire was also evaluated through a convenience sample from dental assistant students (*N* = 39). Content validity was determined by expert consensus, as well as exploratory and confirmatory factor analysis. The total scale demonstrated very good internal consistency (Cronbach’s alpha: 0.71). Reproducibility was also very good, as the kappa coefficient was higher than 0.72 for the majority of items (range: 0.63 to 0.97). The survey took a mean of 17.4 min (±4.3) to administer.

### 2.3. Interviews 

The survey was conducted as an anonymous online self-administered questionnaire sent to each dental practice and was filled out by one of the assistants of the practice. Data were collected from the questionnaires described above. A single questionnaire was filled out per practice (statistical unit) by one of the assistants. 

### 2.4. Variables 

#### 2.4.1. Criteria Studied

The process of sterilization of critical instruments for preventing iatrogenic transmission is divided into 3 steps: packaging, processing with a sterilizing agent, and verification of process efficacy. 

#### 2.4.2. Variables Generated from Indicators 

The conformity to the guidelines was determined for each indicator. For binary variables, a positive answer was expected. When a procedure was described by the frequency of implementation, adherence to the guidelines was considered only when systematically performed (“always”). Percentages were computed after excluding the missing data and the “Not Applicable” category. The “No idea” category was coded as “not in compliance with the guidelines” as DAs are supposed to be aware of all sterilization procedures.

#### 2.4.3. Global Descriptive Indicators

For each group, the sum of indicators was computed in conformity with the guidelines. A dental practice was considered as satisfactorily implementing a given group of procedures when over 80% of them were performed in accordance with the guidelines. 

### 2.5. Analyses

The dental office was the statistical unit of the analyses. First, the descriptive characteristics of the dental practices (number of dental units, dentists and DAs) and the distributions of the 29 indicators were presented in each group. Then, the four global descriptive indicators were studied according to the number of dental units (–2 vs. 3+), sum of dentists + DAs in the practices (2–3; 4–5; 6+) and the DA/dentist ratio (<1; parity; >1).

Adherence to guidelines by practices was focused on their global adherence to the procedures of each group. Global adherence was based on the percentage of procedures correctly performed in each of the procedure groups. It was assumed that the “global adherence” to a given group procedure was “satisfactory” when over 80% of the procedures from the group were performed correctly. Thus, 4 global indicators were dichotomized into over 80% vs. lower than 80%, and these indicators were derived for each procedure group. 

Interrelations between the different binary global indicators were then investigated, using pairwise comparisons, with chi-square tests (Fisher exact tests when validity criteria were not met). The level of agreement complemented these statistical tests. The interpretation of kappa coefficients was based on Cohen’s classification: ≤0, no agreement; 0.01–0.20, poor; 0.21–0.40, fair; 0.41–0.60, moderate; 0.61–0.80, substantial; and 0.81–1.00, almost perfect agreement [29].

## 3. Results

### 3.1. Characteristics of Dental Practices

A total of 179 practices from the Rhône-Alpes region in France were included in the analyses. The number of dental units per practice ranged from 1 to 9, with 2 as the median value (Q25%–Q75% = 2–4). The maximum number of dentists per practice was 10, while the median value was only 2 (Q25%–Q75% = 1.5–3). The corresponding results for the number of assistants were 10, 2 and (Q25%–Q75% = 1–3). When considering the ratio of assistants to dentists, 53.1% of practices presented a parity in the number of dentists and assistants, 23.5% had more dentists than assistants, and vice-versa, for an equal proportion.

### 3.2. Conformity of Sterilization-Related Procedures

The adherence level to sterilization guidelines noticeably varied between dental practices according to the different procedures, from 20.7% to 82.6% (median percentage: 61.6%, Q25%–Q75% = 44.2%–71.1%).

● Group 1: Pre-sterilization cleaning of reusable instruments

Among the six indicators, the proportion of dental practices operating in accordance with the guidelines ranged from 26.3% (V4) to 78.8% (V2), and the median percentage was 58.1%. Only 23.4% of them were in conformity for over 80% of the indicators (5 or 6/6) (Table 1). 

● Group 2: Biological verification of sterilization cycles—Monitoring steam sterilization procedures

Only one indicator out of 9 in this group exceeded 60% for correct implementation of the procedure. The success rate for the others varied from 20.7% for V13 to 62.9% for V11 (median percentage 50.9%). Only 6.6% of them adhered to the guidelines for over 80% of the indicators (Table 2). 

● Group 3: Autoclave performance and practitioner knowledge of autoclave use

Adherence to the recommendations ranged from 57.7% (V23) to 79.4% (V22) of dental practices (median value 69.2%). The prion cycle was properly performed by 68.2% of practices. Overall, 46.6% of practices successfully performed over 80% of the group procedures. 37.1% declared that they do not know how to comply with the norm EN 13060 type B; they only had an autoclave able to sterilize types of load such as dynamic instrumentation, handpieces, surgical suction cannulas, counter angles, surgical instruments and endo cannula instruments (Table 3).

● Group 4: Monitoring and documentation of sterilization procedure—Tracking and tracing the instrumentation

The correct implementation of the different procedures noticeably varied from 43.0% (V27) to 67.1% (V28). The median value was 58.2%. Overall, 38.6% of the dental practices reached the 80% threshold of indicators correctly performed (Table 4).

### 3.3. Stratified Global Indicators According to the Size of Dental Practices and the Relative Percentages of DAs in the Team

The percentage of global implementation of Group 4 procedures (>80%) was twice as high in dental practices with 3 or more dental units compared to the smaller ones (*p* = 0.002). Global adherence to Group 3 procedures was also better when the team included over 6 caregivers, although a U-shaped relationship appeared, with a nearly significant difference. Success of Group 2 procedures’ global implementation was higher in the case of 3+ dental units, although caution is required, given the low percentages in this group. Other associations did not reach significance level, notably with DA/dentist ratio. Likewise, all associations were non-significant for Groups 1 and 3 (Table 5).

### 3.4. Interrelations between Stratified Global Indicators 

● Pairwise comparisons

Significant pairwise associations were noted between global adherence to Group 4 procedures with the successful implementation of all other groups of procedures. There was, proportionally, a 2.5- and 2-fold increase in the successful implementation of Group 4 procedures when Group 3 and Group 4 procedures were properly followed, respectively. Corresponding levels of agreement were considered as “fair” for Group 3 procedures with virtually 75% of concordant practices and “poor” with Group 1 procedures. A significant association emerged between global adherence to Group 2 and Group 3, with a “poor” level of agreement. Conversely, Groups 1, 2 and 3 did not present any significant statistical pairwise associations with one another, while corresponding levels of agreement were all classified as “poor” (Table 6).

Nevertheless, the levels of agreement were only classified as “fair”, while the percentage of concordant practices regarding global adherence towards both groups approached 75% (Table 6). Global adherence to Group 4 procedures also correlated with good results for Group 1 procedures, though less markedly (Table 6). The significant association between correctly implementing Group 2 and Group 4 procedures must be cautiously interpreted, given the low proportion of dental practices globally implementing Group 2 procedures with success (Table 6). Conversely, the quality of autoclave use (Group 3) did not yield any significant association with global correct implementation of pre-sterilization cleaning (Group 1), with a poor level of agreement between both domains. Both groups of procedures were clearly distinguished in the multi-criteria analysis. There was no association with global adherence to biological verifications of cycles and monitoring steam procedures (Group 2), either. Likewise, other pairwise between-group comparisons (1 vs. 2 and 2 vs. 3) were not significant, either (Table 6).

## 4. Discussion

Prions, the infectious agent of CJD, differ from other infectious agents as their infectivity can entail conformational modifications of normal prions [30]. They may not be inactivated by means of routine surgical instrument sterilization procedures [31]. As a consequence, the sterilization of prions requires the denaturation of the protein, resulting in an inactivation of pathological prions, which lose their ability to induce an abnormal folding of normal prions [32]. The high resistance of prions to standard sterilization methods warrants special procedures in the reprocessing of surgical instruments [33].

Despite the emergence of recent studies, evidence remains limited on the quality of implementation of sterilization procedures in dental practices [26]. This is one of the few recent surveys conducted on this topic in French dental practices and specifically focused on sterilization processes of instruments. As a rule, the overall adherence level to procedures was unsatisfactory for most of the 179 dental practices. The percentages of practices correctly performing over 80% of the procedures ranged from 6.6% to 45.7% in the four groups.

Some differences were noted between procedure groups. While all procedures in Group 3 were properly implemented by over 60% of practices, virtually none were in Group 2, which presented the highest failure rate regarding adherence to the guidelines. Despite these differences, our findings suggest that adherence to the different sterilization procedures remains globally inadequate in the majority of dental practices. The best global implementation of procedure groups did not reach the 50% threshold (46.6% in Group 3). Our worrisome results align with those of preceding studies [34,35,36]. 

This unsatisfactory situation could possibly be explained by several factors. Firstly, an inadequate knowledge or understanding of some detailed procedures should not be overlooked. Surprisingly, the “No idea” reply did not appear frequently in our results. Other reasons could be a lack of motivation or practical organizational issues, difficulty in complying with or understanding the guidelines [37,38], practical organizational issues, or merely lack of time [39]. 

Non-employer practitioners report that they cannot be used for financial reasons. For dentists who have employed receptionists, 21% say they entrust their receptionists with performing sterilization. These tasks are not the legal responsibility of a receptionist. (Non-employer practitioners report that they find it impossible to employ appropriate staff for financial reasons. Consequently, 21% of employers of receptionists state that they entrust the latter with the task of sterilization. However, this task is not part of a receptionist’s legal job duties). In France, the personnel qualified to work in the dental office are mainly aide assistants and dental assistants. Receptionists and secretaries, as their name indicates, are strictly assigned to administrative and reception tasks and are therefore not authorized to provide medical assistance with regard to sterilization, preparation of equipment and assistance.

Interestingly, some factors influencing adherence level to sterilization procedures have been identified, such as the number of daily patients, dentist age and/or gender [40], and the level of information received by dental caregivers on iatrogenic infectious risk [39,41], but further evidence is needed. 

As a rule, correct performance of global indicators did not significantly vary with the dental practice-related factors studied, except for Group 4, wherein results were significantly better in practices with 3 or more dental units. A significant impact of the size also emerged for Group 2, though interpretation requires caution due to the low percentage of success (6.6%). Better adherence to sterilization procedures in larger practices has been described [42]. Bigger practices could be endowed with more resources in terms of space and available staff. Surprisingly, an impact of size of practices was not seen in Groups 1 and 4. Additionally, the DA/dentist ratio seemed to have a more limited influence. Overall, these data suggest that improvement in adherence to procedures is desirable for most dental practices, even among those of bigger size.

Some limitations should be borne in mind. Regions in France have considerable discretionary power over infrastructural spending, e.g., education, health, universities and research, and assistance to business owners. This has meant that the heads of wealthy regions such as Rhône-Alpes can be high-profile positions. This is a restrictive criterion in our study that must be noted. In our study, a single DA of the practice completed the questionnaire on behalf of the whole team. Thus, we have no guarantee that identical replies would have been provided by any other DAs in practices with 2 or more DAs. We specifically focused on the indicators of sterilization for reusable instruments. The reusable critical instruments classified as being at high risk of infection primarily include invasive instrumentation (sensors, ... curette), and must be sterilized and kept sterile between each use. Other components of prevention, such as the presence of a dedicated area for the instrument cleaning, disinfections of surfaces, systematic hand washing, changing gloves after each patient, management of waste disposal, and water lines were not covered by the present study [43,44]. As our data originate from self-administered questionnaires, their validity may not be optimal, with a possible overestimation of adherence due to desirability bias, namely failure to report inadequate implementation of procedures. Our study sample, given the limited response rate, was not representative of the overall dental practices in France. Indeed, all of those enrolled in our study were endowed with one DA or more, whereas nearly half of dental practices have none at the national level. For these reasons, the actual adherence rate can reasonably be assumed to be even more alarming in a more representative sample or in French dental practices overall.

The present study bears implications both in terms of research and public health, given the risk of CJD infection contamination. Our findings underscore the need to strengthen the education of dentists and DAs toward improved implementation of procedures regarding sterilization of instruments. For better efficiency, a critical preliminary step should be the comprehensive investigation of the reasons why the different recommended procedures are inadequately performed by dental practices. Qualitative studies could contribute to identify these reasons, and particularly the different barriers encountered by dental practices. Before implementing any educational campaign, in view of choosing the optimal approach, it should be verified to which extent non-adherence to the different groups of procedures are intercorrelated with one another. It is crucial for all dental students to be up to date on current guidelines, equipment, and techniques for proper infection control. The gap found in our study between current scientific knowledge of the management of sterilization and their implementation in dental practices must challenge us. Identifying the cause of malfunctions should allow the necessary implementation of corrective and preventive measures. 

However, education limited to a single session may not be sufficient to bring about any perennial change in daily behaviors. It does appear that an implementation strategy is required to encourage the implementation of the decontamination guidance [38]. Long-term regular training sessions could be useful, particularly in the case of deficient knowledge and/or awareness or motivation. Better adherence levels to sterilization procedures were consistently found when practices underwent continuous education [45,46], notably on prion contamination risk [47]. More generally, regular assessments of the quality of implementation of the different sterilization procedures are highly desirable at the national or regional level to monitor these public health issues on a regular basis.

## 5. Conclusions

In conclusion, the practitioner is obligated to provide results regarding sterilization. The practitioner, and no one else, is responsible for health safety and for infectious risks in his dental office. He is responsible for permanently establishing proof of his actions. These standard recommendations—simple, basic—may reduce the risk of CJD infections during care, but they must be impeccable in their implementation. Following the discovery of the tasks not being carried out according to the guidelines in force, it is urgent to anticipate and propose alternative measures, compulsory or not, for the near future.

## Figures and Tables

**Table 1:** Descriptive analysis: Conformity with sterilization-related procedures (Group 1) (*N* = 179).

Practice Items	*N*	%	% Conform to the Guidelines
**V1 All medical devices are packaged in welded bags**			75.42
Yes	135	75.42	
No	44	24.58	
**V2 All autoclavable medical devices are autoclaved**			78.77
Always	141	78.77	
Often	16	8.94	
Sometimes	10	5.59	
Seldom	10	5.59	
Never	2	1.12	
**V3 All detachable instruments (turbines, etc.) are removed after each treatment, and processed separately**			35.20
Always	63	35.20	
Often	19	10.61	
Sometimes	21	11.73	
Seldom	61	34.08	
Never	15	8.38	
**V4 Instruments are dried manually and carefully, using a single-use non-woven cloth and/or filtered compressed air**			26.25
Always	47	26.25	
Often	7	3.91	
Sometimes	9	5.03	
Seldom	80	44.69	
Never	11	6.15	
Not applicable	25	13.97	
**V5 All pre-sterilization stages recommended by the manufacturer are followed**			58.10
Always	104	58.10	
Often	15	8.38	
Sometimes	1	0.56	
Seldom	5	2.79	
Never	54	30.17	
**V6 Inoperable custom-made devices are disposed of as healthcare waste**			43.58
Yes	78	43.58	
No	8	4.47	
No idea	93	51.96	
**Sum of procedures in accordance with Guidelines (*N* = 154)**			Over 80% compliant behaviors: 23.38 (100%: 9.74)
0	10	6.49	
1	20	12.99	
2	22	14.29	
3	32	20.78	
4	34	22.08	
5	21	13.64	
6	15	9.74	

**Table 2:** Descriptive analysis: Biological verification of sterilization cycles—Monitoring steam sterilization procedure (Group 2) (*N* = 179).

Practice Items	*N*	%	% Conform to the Guidelines
**V7 Update of device journal**			41.90
Yes	75	41.90	
No	104	58.10	
**V8 Autoclaves are suitable for reprocessing critical medical devices**			43.58
Yes	78	43.58	
No	6	3.35	
No idea	95	53.07	
**V9 Repeat reprocessing protocol is used in the event of failure**			52.51
Always	94	52.51	
Often	9	5.03	
Sometimes	5	2.79	
Seldom	9	5.03	
Never	37	20.67	
No idea	25	13.97	
**V10 Compliance of load release protocol**			49.41
Always	84	49.41	
Often	10	5.88	
Sometimes	5	2.94	
Seldom	5	2.94	
Never	16	9.41	
No idea	45	26.47	
Not applicable	5	2.94	
**V11 The stream sterilizer complies with standard EN 13060**			62.94
Yes	107	62.94	
No idea	63	37.06	
**V12 The personnel tasked with processing medical devices receives specific training, updated regularly**			36.87
Yes	66	36.87	
No idea	113	63.13	
**V13 There is a document in which all the events affecting the autoclave are noted (servicing, maintenance, breakdowns, etc.)**			20.67
Yes	37	20.67	
No	142	79.33	
**V14 Information provided by the manufacturers mention that rotary instrument holders (turbines, contra-angles and handpieces) are ready to be pre-disinfected, cleaned and sterilized**			53.50
Always	84	53.50	
Often	14	8.92	
Sometimes	2	1.27	
Seldom	3	1.91	
Never	5	3.18	
No idea	41	26.11	
**V15 After-sales service for the sterilizer is performed**			53.25
Yes	82	53.25	
No	17	11.04	
No idea	55	35.71	
**Sum of procedures in accordance with the Guidelines (*N* = 135)**			Over 80%: 6.67 (100%: 0.74)
0	7	5.19	
1	7	5.19	
2	19	14.07	
3	13	9.63	
4	18	13.33	
5	25	18.52	
6	15	11.11	
7	22	16.30	
8	8	5.93	
9	1	0.74	

**Table 3:** Descriptive analysis: Autoclave performance and practitioner knowledge of autoclave use (Group 3) (*N* = 179).

Practice Items	*N*	%	% Conform to the Guidelines
**V16 Compliant implementation of prion cycle**			68.21
Yes	118	68.21	
No	55	31.79	
**V17 Handling, loading, monitoring**			76.30
Always	132	76.30	
Often	30	17.34	
Sometimes	4	2.31	
Seldom	2	1.16	
Never	2	1.16	
No idea	2	1.16	
Not applicable	1	0.58	
**V18 When loading the steam sterilizer, the manufacturer’s recommendations are followed, or failing this, the bagged devices positioned are standing on edge, with paper touching paper and plastic touching plastic, without touching the walls, and not too tightly together**			66.04
Always	105	66.04	
Often	23	14.47	
Sometimes	6	3.77	
Seldom	9	5.66	
Never	2	1.26	
No idea	7	4.40	
Not applicable	7	4.40	
**V19 Temperature and duration of sterilization tray**			70.32
Always	109	70.32	
Often	9	5.81	
Sometimes	1	0.65	
Seldom	5	3.23	
Never	17	10.97	
No idea	9	5.81	
Not applicable	9	5.81	
**V20 Compliant result obtained for physicochemical integrator packaged inside bags and placed within the load**			65.16
Always	101	65.16	
Often	6	3.87	
Sometimes	4	2.58	
Seldom	3	1.94	
Never	19	12.26	
No idea	9	5.81	
Not applicable	13	8.39	
**V21 Result obtained for all flow indicators appearing on bags**			68.39
Always	106	68.39	
Often	7	4.52	
Sometimes	3	1.94	
Seldom	2	1.29	
Never	18	11.61	
No idea	10	6.45	
Not applicable	9	5.81	
**V22 Absence of moisture in bags and packaging integrity**			79.35
Always	123	79.35	
Often	9	5.81	
Sometimes	3	1.94	
Seldom	1	0.65	
Never	7	4.52	
No idea	6	3.87	
Not applicable	6	3.87	
**V23 Result of the last steam penetration test (Helix device)**			57.69
Always	90	57.69	
Often	11	7.05	
Sometimes	7	4.49	
Seldom	9	5.77	
Never	22	14.10	
No idea	8	5.13	
Not applicable	9	5.77	
**Sum of procedures in accordance with the Guidelines (*N* = 131)**			Over 80%: 46.57 (100%: 22.14)
0	1	0.76	
1	4	3.05	
2	1	0.76	
3	10	7.63	
4	14	10.69	
5	20	15.27	
6	20	15.27	
7	32	24.43	
8	29	22.14	

**Table 4:** Descriptive analysis: Monitoring and documentation of sterilization procedure—Tracking and tracing the instrumentation (Group 4) (*N* = 179).

Practice Items	*N*	%	% Conform to the Guidelines
**V24 Quality of verification ensuring successful completion of cycle**			51.96
Yes	92	51.96	
No	57	31.28	
No idea	30	16.76	
**V25 Information required for traceability is entered in the I.T. system**			58.23
Yes	92	58.23	
No	52	32.91	
No idea	14	8.86	
**V26 I.T. system connects the medical device to the patient**			45.86
Yes	72	45.86	
No	59	37.58	
No idea	26	16.56	
**V27 Monthly monitoring of sterilization using biological indicators**			43.05
Yes	65	43.05	
No	45	29.80	
No idea	41	27.15	
**V28 Monitoring of sterilization using flow indicators**			67.11
Yes	102	67.11	
No	21	13.82	
No idea	29	19.08	
**V29 Monitoring of sterilization using integrators**			63.58
Yes	96	63.58	
No	22	14.57	
No idea	33	21.85	
**Sum of procedures in accordance with the Guidelines (*N* = 140)**			Over 80% compliant behaviors: 38.57 (100%: 22.86)
0	15	10.71	
1	11	7.86	
2	19	13.57	
3	23	16.43	
4	18	12.86	
5	22	15.71	
6	32	22.86	

**Table 5:** Global adherence (>80%) to the four procedure groups ^(1)^ according to the characteristics of dental practices.

Global Indicators		>80% of Compliant Procedures in Group 1, *N* = 154		>80% of Compliant Procedures in Group 2, *N* = 135		>80% of Compliant Procedures in Group 3, *N* = 129		>80% of Compliant Procedures in Group 4, *N* = 140	
		%	*p*-Value ^(2)^	%	*p*-Value ^(2)^	%	*p*-Value ^(2)^	%	*p*-Value ^(2)^
**# of caregivers (dentists + dental assistants)**	2–3	27.3	0.44	2.3	0.34 *	58.3	0.07	37.8	0.05
	4–5	17.3		7.7		33.3		25.0	
	6+	25.9		9.6		49.1		49.1	
	Total	23.4		6.6		46.6		38.6	
**# of dental units**	1–2	21.9	0.65	1.4	0.01 *	43.1	0.42	25.8	0.002
	3+	25.0		12.3		50.0		51.4	
	Total	23.4		6.6		46.6		38.6	
**Ratio # of dental assistants/ # of dentists**	<1	16.2	0.23	6.9	0.87 *	45.2	0.98	32.3	0.63
	Parity	22.6		5.7		50.0		39.1	
	>1	33.3		8.3		47.1		43.2	
	Total	23.4		6.6		46.6		38.6	

^(1)^ Group 1: Pre-sterilization cleaning of reusable instruments; Group 2: Biological verification of sterilization cycles—Monitoring steam sterilization procedures; Group 3: Autoclave performance and practitioner knowledge of autoclave use; Group 4: Monitoring and documentation of sterilization procedure—Tracking and tracing the instrumentation; ^(2)^ Chi-square test or Fisher test in case of asterisk (*); #: Number in case of bookmark.

**Table 6:** Pairwise statistical relationships and levels of agreement between global adherence to procedures (>80%) in the different groups ^(1)^.

Global Indicators		>80% of Compliant Procedures Group 2			>80% of Compliant Procedures Group 3			>80% of Compliant Procedures Group 4		
		%	*p*-Value ^(2)^	Kappa ^(3)^ % Agreement	%	*p*-Value ^(2)^	Kappa ^(3)^ % Agreement	%	*p*-Value ^(2)^	Kappa ^(3)^ % Agreement
**>80% of compliant procedures Group 1**	>80%	10.7	0.17 * (*n* = 111)	κ = 0.096 74.8 Poor	62.5	0.22 (*n* = 112)	κ = 0.19 60.7 Poor	60.0	0.003 (*n* = 117)	κ = 0.26 67.5 Fair
	≤80%	3.6			40.0			29.9		
	Total	5.4			46.4			37.6		
**>80% of compliant procedures Group 2**	>80%				66.7	0.30 * (*n* = 117)	κ = 0.06 55.6 Poor	77.8	0.03 * (*n* = 127)	κ = 0.13 63.8 Poor
	≤80%				45.4			37.3		
	Total				47.0			40.2		
**>80% of compliant procedures Group 3**	>80%							62.1	<0.0001 (*n* = 121)	κ = 0.37 68.6 Fair
	≤80%							25.4		
	Total							43.0		

^(1)^ Group 1: Pre-sterilization cleaning of reusable instruments; Group 2: Biological verification of sterilization cycles—Monitoring steam sterilization procedures; Group 3: Autoclave performance and practitioner knowledge of autoclave use; Group 4: Monitoring and documentation of sterilization procedure—Tracking and tracing of the instrumentation; ^(2)^ Chi-square test or Fisher test in case of asterisk (*); ^(3)^ Classification of level of agreement based on Cohen’s classification of Kappa values: ≤ 0 as indicating no agreement and 0.01–0.20 as none to slight, 0.21–0.40 as fair, 0.41–0.60 as moderate, 0.61–0.80 as substantial, and 0.81–1.00 as almost perfect agreement.
